
JOURNAL INFORMATION
==============================
NLM Title Abbreviation: Atlanta J Rec Med
NLM Journal ID: 0001011

Atlanta Journal-Record of Medicine

Publisher: Biomedical Journal Digitization Project

ARTICLE INFORMATION
==============================
PMCID: PMC9038283
Article version: 1
Subjects: Book Reviews

Book Reviews

Print publication date: 1914 Dec
Volume: 61
Issue: 9
First page: 445
Last page: 446
License: This work is free of known copyright restrictions. For more information, please see PMC Back Issue Digitization.
License URL: https://creativecommons.org/publicdomain/mark/1.0/

==============================

IF YOUR CHOICE OF HYPNOTICS
in the presence of a distinct valvular lesion or a myocardial weakness, has been
PASADYNE
(DANIEL'S CONCENTRATED TINCTURE OF PASSIFLORA INCARNATA.)
you may well know that you have carefully observed the "safety first" rule.
Although of the highest efficiency in the relief of sleeplessness and nervousness PASADYNE (Daniel) is without immediate or remote evil after-effects.
SAMPLES AND LITERATURE SUPPLIED TO PHYSICIANS PAYING EXPRESS CHARGES.
Laboratory of JOHN B. DANIEL, Atlanta, Ga.

perfectly that supplication to the deity which admits with shame "there is no health in us."

If war is a necessary consequence of overpopulation, it is time then for man to see to it that overpopulation does not occur again. He has overcome the force of gravity in a myriad ways; he has overcome the darkness of night—-electricity does his bidding in the remotest corners of the earth—he sails upon the sea and through the air, he has conquered almost all the forces of nature, but to what end if he does not conquer himself?

Fortunately, even in the most prolific countries, birth rates are beginning to fall, and presently a low birth rate will be one of the marks of culture in a nation as it now is in classes and families.—Lancet Clinic.

### BOOK REVIEWS

THE CLINICS OF JOHN B. MURPHY, M. D., at Mercy Hospital, Chicago. Volume III, Number III. Octavo of 215 pages, 54 illustration. Philadelphia and London: W. B. Saunders Company, 1914. Published Bi-Monthly. Price per year: Paper, $8.00; Cloth, $12.00.

Murphy presents in a terse, condensed manner the essential facts of modern surgery.

PROGRESSIVE MEDICINE. A quarterly digest of advances, discoveries and improvements in the medical and surgical sciences. Edited by Hobart Amory Hare, M. D. Professor of Therapeutics, Materia Medica and Diagnosis in the Jefferson Medical College, Philadelphia; Physician to the Jefferson Medical College Hospital; One Time Professor of Diseases of Children in the University of Pennsylvania; Member of the Association of American Physicians, Etc., assisted by Leighton F. Appleman, M. D., Instructor in Therapeutics, Jefferson Medical College, Philadelphia; Ophthalmologist to the Frederick Douglas Memorial Hospital; Instructor in Ophthalmology, Philadelphia Polyclinic Hospital and College for graduates in medicine. Volume 2, June, 1914, Hernia, Surgery of the Abdomen, exclusive of Hernia, Gynecology, diseases of the blood, Diathetic and Metabolic Diseases. Diseases of the spleen, thyroid gland, nutrition and the Lymphatic system. Ophthalmology. Lea and Febiger, Philadelphia and New York. 1914.

As usual this excellent digest contains a vast amount of valuable facts.

---
